# HbSC Disease and Spontaneous Epidural Hematoma with Kernohan's Notch Phenomena

**DOI:** 10.1155/2015/470873

**Published:** 2015-10-21

**Authors:** Meera Yogarajah, Chidozie Charles Agu, Bhradeev Sivasambu, Mark A. Mittler

**Affiliations:** ^1^Department of Medicine, Interfaith Medical Center, Brooklyn, NY 11213, USA; ^2^Department of Neurosurgery, North Shore-LIJ Health System, Manhasset, NY 11030, USA

## Abstract

Spontaneous (nontraumatic) acute epidural hematoma is a rare and poorly understood complication of sickle cell disease. A 19-year-old African American male with hemoglobin SC disease (HbSC) presented with generalized body aches and was managed for acute painful crisis. During his hospital stay he developed rapid deterioration of his mental status and computed topography revealed a spontaneous massive epidural hematoma with mass effect and midline shift with Kernohan's notch phenomena for which urgent craniotomy and evacuation was done. We report the first case of HbSC disease associated with catastrophic epidural hematoma progressing to transtentorial herniation and Kernohan's notch phenomena within few hours with rapid clinical deterioration. The etiopathogenesis and the rare presentation are discussed in detail in this case report.

## 1. Background

Sickle cell disease is associated with cerebrovascular complications and the risk varies with the genotype. The Cooperative Study of Sickle Cell Disease revealed a significantly high incidence of cerebrovascular events in HbSS disease at 0.61 per 100 patient-years with corresponding low values of 0.15, 0.09, and 0.08, respectively, for HbSC, HbS-beta (+) thalassemia, and HbS beta (0) thalassemia [1]. Intracranial hemorrhage constitutes one-third of the cerebrovascular event in sickle cell disease with higher incidence noted in young adults as opposed to cerebral infarction which occurs more in children. Subarachnoid, intraparenchymal, intraventricular, or a combination of these hemorrhages is commonly associated with sickle cell disease. Spontaneous epidural hematoma is a rare poorly understood complication of sickle cell disease.

## 2. Case Presentation

**Figure 1:**
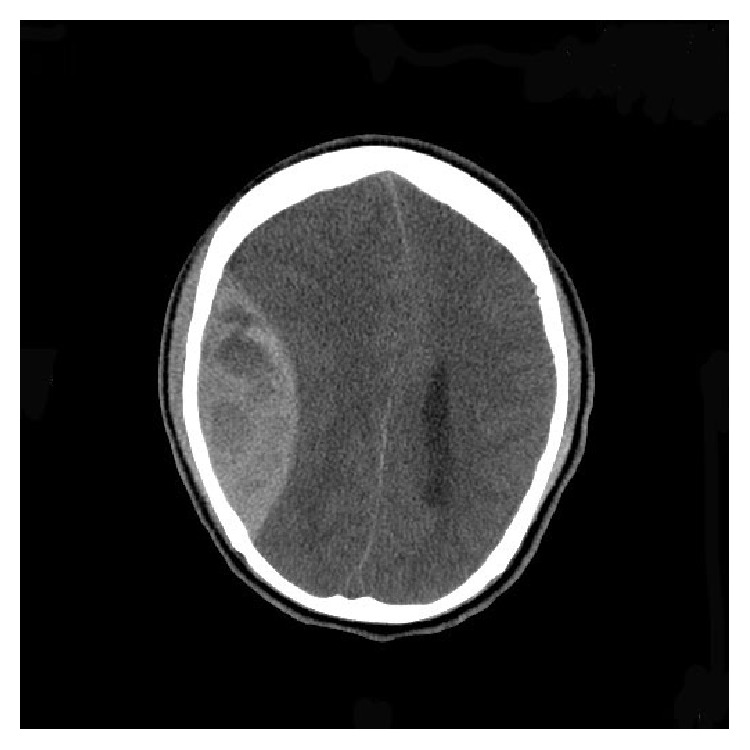
Preop CT showing right large epidural hematoma with midline shift.

**Figure 2:**
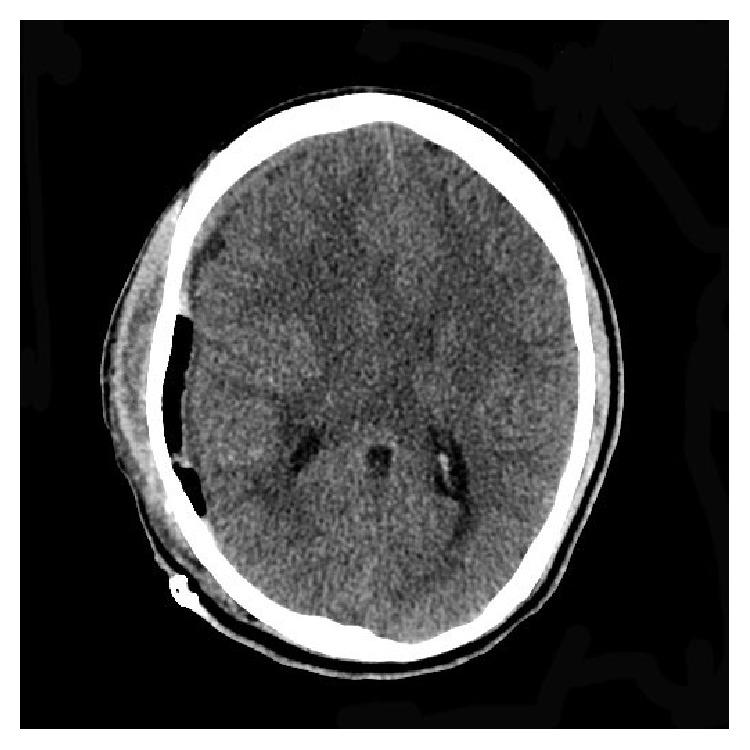
Postop CT showing resolution of epidural hematoma and postop changes on right.

**Figure 3:**
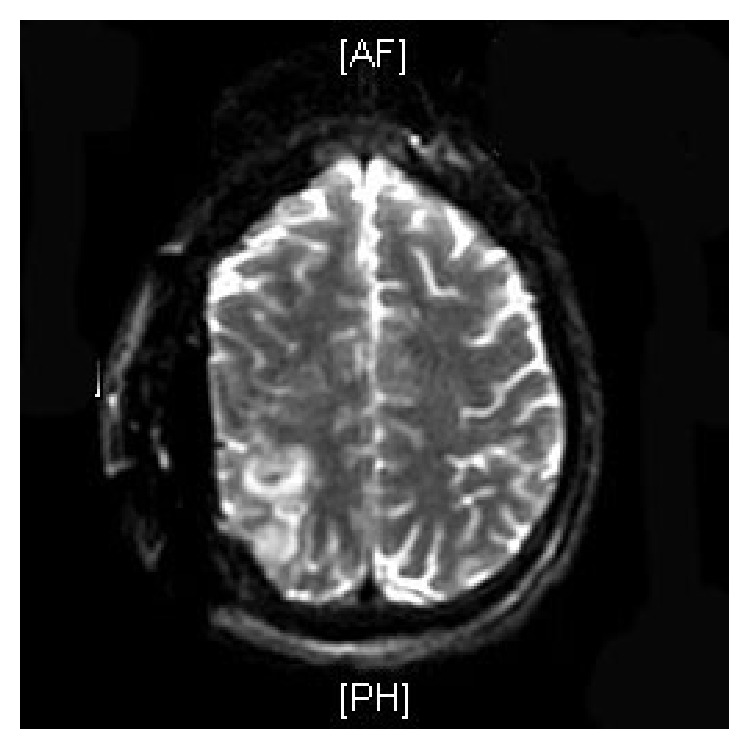
Diffusion weighted magnetic resonance image demonstrating right parietal infarct.

**Figure 4:**
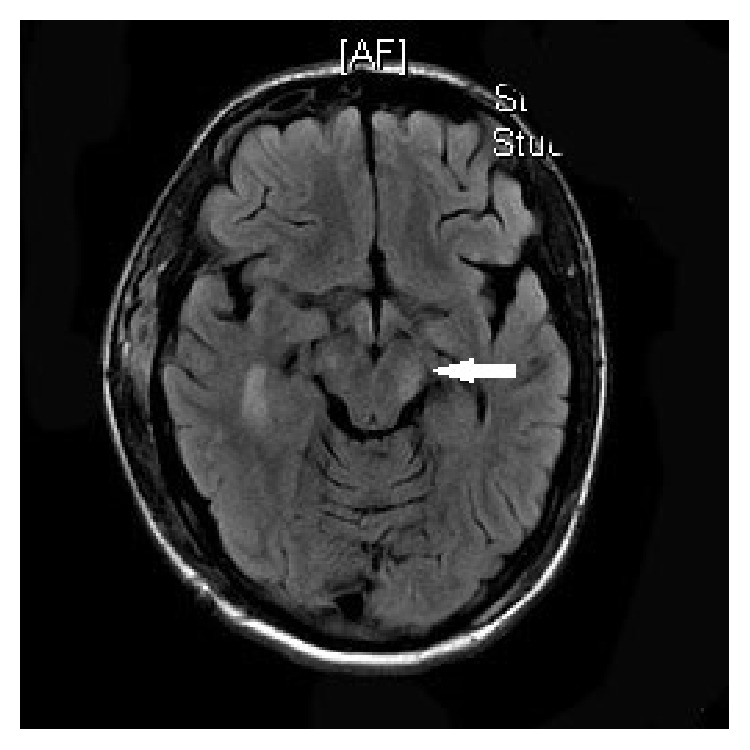
Postop MR demonstrating contralateral (left) cerebral crus infarct from herniation (Kernohan's notch phenomenon).

**Figure 5:**
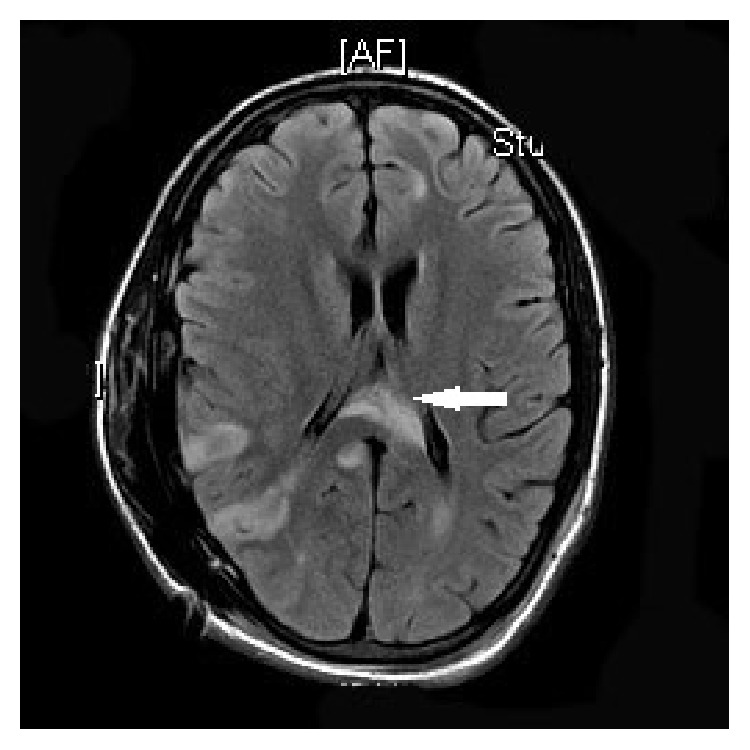
Postop MR demonstrating infarcts from herniation including the left cerebral crus infarcts (white arrow) and right parietal infarcts.

A “19-year-old” African American male with HbSC disease presented with generalized body aches for “4 hours' duration” which did not respond to ibuprofen which he usually uses for pain. He denied any other systemic complaints and was unable to identify any trigger factors for his painful crisis. He had multiple inpatient admissions in the past for painful crisis but did not have any history of previous stroke, acute chest syndrome, or other sickle cell disease associated complications. On examination patient was afebrile and hemodynamically stable with a blood pressure of 121/83 mmhg and pulse rate of 63/min. Systemic examination was unrevealing. Initial labs revealed white blood cell count of 10.3 k/uL, hemoglobin of 12.7 g/dL, hematocrit of 39.4%, and platelet count of 177 k/uL. The electrolytes, renal function test, and liver enzymes were normal except for mild hyperbilirubinemia of 2.2 mg/dL (0.3–1.2). His coagulation panel was normal. Patient was managed for acute vasoocclusive crisis with intravenous hydration and opioids optimized according to the pain scale. Patient continued to have generalized body aches during the hospital stay and his opioids were increased after which he was comfortable. On the second day of admission patient suddenly became diaphoretic and unresponsive. Naloxone was administered with improvement in mentation; however, in 3 hours patient again became drowsy and was only responding to deep painful stimuli. Emergent computed topography of the head revealed large 3.6 × 9.7 cm right acute epidural hematoma with mass effect on the adjacent sulci and right lateral ventricle with 1.6 cm right-to-left midline shift (Figure 1). There was no history of trauma prior to hospitalization and he did not sustain any trauma during the hospital stay. Patient underwent urgent craniotomy and evacuation of the epidural hematoma (Figure 2). There was no evidence of skull infarction and the bony margins were regular and not thickened with no periosteal elevation and a bleeding vessel was not identified. On recovery patient had mild ipsilateral hemiparesis and postoperative imaging revealed Kernohan's notch phenomena (Figures 3, 4, and 5).

## 3. Discussion

Epidural hematoma commonly occurs secondary to trauma with associated skull fracture and arterial injury in 85% of the cases [2, 3] and the damage to the dural sinuses in 15% of the cases [2]. Spontaneous epidural hematoma by itself is rare. Spontaneous epidural hematoma secondary to sickle cell disease has been scarcely described in the literature and the mechanism still remains obscure. Skull infarction causing periosteal elevation and disrupting the cortical bone and eventually bleeding into the epidural place is one of the proposed mechanisms [4]. Another postulation is inadequate venous drainage causing edema and triggering bleeding due to rupture of the veins [5]. Though extra medullary hematopoiesis commonly occurs in long bones, it rarely occurs in the calvarium and during acute hemolytic crisis expansion of the skull bone due to hematopoiesis can disrupt the bony margins and predispose to rupture of the vessels and bleeding [6]. The pathogenesis of bleeding in our patient was not identified as there was no evidence of bone infarction and no bleeding vessel was noted. The possible explanation as described in the literature by Dahdaleh et al. would be disruption of the cortical bone margin occurring at a microscopic level [6]. Low steady-state hemoglobin and high steady-state leucocyte count have been associated with increased propensity for hemorrhagic stroke in sickle cell disease [1]. However our patient did not have any of these risk factors. Other postulated risk factors include previous ischemic stroke, moyamoya lesions [7], recent hypertension, transfusion, and treatment with steroids [8]. Spontaneous epidural hematoma has also been reported with infections like sinusitis and epidural abscess, coagulopathy, chronic kidney disease and hemodialysis, metastasis to the dura, and vascular malformations [9, 10].

The classical lucid interval that occurs in traumatic epidural hematoma due to concussion of the brain does not occur in spontaneous (nontraumatic) epidural hematoma. Headache is the commonest clinical manifestation mostly reported secondary to epidural hematoma associated with sickle cell disease; however, our patient only complained of generalized worsening body aches and the opioid dose was increased which likely masked the initial warning signs of epidural hematoma.

The literature review revealed few reported cases of epidural hematoma in association with sickle cell disease which occurred predominantly with HbSS genotype [4–6, 10]. HbSC disease is less commonly associated with cerebrovascular accidents and there has been only one reported case of epidural hematoma associated with HbSC disease [11]. The etiology of the epidural hematoma in this patient was skull infarction and patient had a small adjacent epidural hematoma which was managed conservatively. We report the first case of HbSC disease associated with catastrophic epidural hematoma progressing to transtentorial herniation and Kernohan's notch phenomena within few hours with rapid clinical deterioration.

## 4. Conclusion

Sudden unresponsiveness in a sickle cell disease patient should prompt the physicians on the possibility of a cerebrovascular complication. Response to Naloxone may be subjective and usually most of these patients are on high doses of opioids to control pain which might mask the initial clinical symptoms and thus may warrant neuroimaging irrespective of the transient improvement of mentation to prevent major sequelae.
